# In this month’s *Bulletin*

**DOI:** 10.2471/BLT.15.000315

**Published:** 2015-03-01

In the editorial section, Sulaiman Bah (134) discusses the possibility of dropping door-to-door enumeration in the next South African census. Priya Agrawal (135) points to persistent challenges in further reductions of maternal deaths in the United States of America.

Gary Humphreys (138–139) reports on the introduction of digital bidding to medicine procurement processes in African countries. Fiona Fleck interviews Eric Goosby (140–141), the United Nations Secretary General’s Special Envoy on Tuberculosis, about efforts to deliver more effective services.

## Uganda

## Bringing malaria tests to market

Jessica Cohen et al. (142–151) measure the effects of stocking rapid diagnostic tests in local drug shops.

## China

## Preventing opportunistic infections

Wei Cheng et al. (152–160) study the effects of adding cotrimoxazole prophylaxis to antiretroviral therapy for HIV.

## Burns, falls and drowning

Li Li et al. (169–175) examine laws designed to prevent childhood injuries.

## Hidden harms

Xiangming Fang et al. (176–185) establish national estimates of child maltreatment.

## Chile

## Reciprocity in organ donation

Alejandra Zúñiga-Fajuri (199–202) reviews a policy change designed to increase the number of organs available for transplant.

## Global

## How long did you wait to see the doctor?

Willemijn LA Schäfer et al. (161–168) assess patient satisfaction with primary care services in 34 countries.

## Seeking informed consent

Nguyen Thanh Tam et al. (186–198) analyse thirty years’ of evidence on informed consent in clinical trials.

## Bringing the data to bear

Rosemary Wyber et al. (203–208) describe potential health-sector use of large quantities of data generated for other purposes.

